# Evaluation of Neuronal Apoptosis Precursors in an Experimental Model of Acute Normovolemic Hemodilution

**DOI:** 10.1371/journal.pone.0108366

**Published:** 2014-09-25

**Authors:** Fabrício O. Frazilio, Denise Aya Otsuki, Jessica Noel-Morgan, Jessica Ruivo Maximino, Gabriela Pintar Oliveira, Gerson Chadi, Jose Otavio Costa Auler, Denise Tabacchi Fantoni

**Affiliations:** 1 LIM08-Laboratory of Anesthesiology, Department of Anesthesia and Surgical Intensive Care, Faculdade de Medicina da Universidade de São Paulo, São Paulo, Brazil; 2 LIM45-Neuroregeneration Center, Department of Neurology, Faculdade de Medicina da Universidade de São Paulo, São Paulo, Brazil; Queen's University Belfast, United Kingdom

## Abstract

**Background:**

The effects of acute anemia on neuronal cells and the safe limits of hematocrit are not well established. The objective of this study was to evaluate neuronal pro- and anti-apoptotic Bax and Bcl-x proteins, caspase-3 and -9 activity, and DNA fragmentation after acute normovolemic hemodilution (ANH).

**Methods:**

Twenty-four pigs were anesthetized and randomized into 4 groups: Sham, ANH to 15% hematocrit (ANH15%), ANH to 10% hematocrit (ANH10%) and hypoxia (Hx). ANH was achieved by simultaneous blood withdrawal and hydroxyethyl starch infusion. Hx consisted of ventilation with a 6% inspired oxygen fraction for 60 minutes. Bax and Bcl-x proteins as well as DNA fragmentation were evaluated in cortical nuclear and mitochondrial fractions. Caspase-3 and -9 activity was evaluated in the cortical mitochondrial and hippocampal cytosolic fractions. The data were compared using analysis of variance followed by Tukey’s test (P<0.05).

**Results:**

No changes were observed in Bax protein expression after hemodilution in the ANH15% and ANH10% groups compared to the Sham group. Bax expression in the Hx group was increased in the nuclear and mitochondrial fractions compared to all other groups. No significant difference was observed in Bcl-x expression. Caspase-3 and -9 activity in the cytosolic and mitochondrial fractions was different in the Hx group compared to all other groups. No statistical significance in DNA fragmentation was found among the Sham, ANH15% or ANH10% groups.

**Conclusion:**

ANH to 10 and 15% hematocrit did not induce alterations in apoptosis precursors, suggesting that cerebral oxygenation was preserved during these anemic states.

## Introduction

Profound acute normovolemic hemodilution (ANH) has been associated with neurological functional alterations as a deficit in cognitive function and memory in experimental [1] and clinical settings with both healthy volunteers and surgical patients submitted to cardiopulmonary bypass (CPB) [2]–[4].

Previous studies have demonstrated that ANH elicits a number of different proteins in the brain such as hypoxia inducible factor-1α, vascular endothelial growth factor, neuronal and inducible nitric oxide synthase. [5], [6] A study with microarray analysis and ANH has also demonstrated an up-regulation of numerous genes involved in inflammatory response: angiogenesis, vascular homeostasis, and apoptosis [7].

Among the several mechanisms that contribute to cognitive impairment, the role of hypoxia-induced neuronal apoptosis has been studied in various scenarios. One of the key neuronal death pathways is mitochondrial, through the activation of apoptotic Bcl-2 family proteins. [8] The neuronal apoptotic pathway has been related to hypoxic injury in several neonatal hypoxia and CPB associated hemodilution studies [9]–[12]; however, no study has investigated the role of these apoptotic proteins in severe acute anemia alone.

This study aimed to test the hypothesis that acute normovolemic anemia with a hematocrit of 10 or 15% could induce the activation of the neuronal mitochondrial-mediated intrinsic apoptotic pathway by evaluating different cellular events involved in hypoxia-induced apoptosis and analyzing some key proteins such as Bax, Bcl-x, caspase 3 and 9, and the presence of endonucleolytic DNA fragmentation, a hallmark of apoptosis, in a porcine model of acute anemia.

## Materials and Methods

### Animals

The study protocol was approved by the Institutional Ethics and Animal Investigation Committee (Comissão de Ética para Analise de Projetos de Pesquisa do HCFMUSP – CAPPesq/0202/08) and was performed in accordance with the Guide for Care and Use of Laboratory Animals [13].

Twenty-four Landrace and Large White crossbreed pigs [30 to 35 kg] were fasted for 12 hours with free access to water. The animals underwent a clinical examination prior to the study and those with any sign of abnormalities were excluded.

### Anesthesia

The animals were sedated with midazolam (0.25 mg. kg^−1^) and ketamine (5 mg. kg^−1^) administered intramuscularly. Anesthesia was induced with propofol (5 mg. kg^−1^ i.v.) and maintained with 1.3 V% isoflurane in oxygen (40%) after endotracheal intubation. Mechanical ventilation (Primus, Dräger, Lübeck, Germany) was volume-controlled, with a tidal volume of 8 mL. kg^−1^ and a respiratory rate set to maintain end-tidal CO_2_ (EtCO_2_) at 40 mmHg. The animals’ temperatures were maintained at approximately 38°C (normal for the species) using warming blankets (Medi-therm II, Gaymar Industries, Orchard Park, NY, USA). All animals received lactated Ringer solution (5 mL. Kg^−1^. h^−1^) as a maintenance fluid.

### Animal preparation and experimental procedures

After the stabilization of an adequate anesthesia depth determined by the absence of an autonomic response to surgical stimuli, catheters were inserted through peripheral cut-downs in the left and right femoral arteries and veins and in the right jugular vein to enable blood withdrawal, fluid replacement, successive collections of blood samples and hemodynamic measurements.

A pulmonary artery catheter (744 H-7.5F, Baxter Healthcare Corporation, Irvine, USA) was inserted in the pulmonary artery trunk with the guidance of typical waveforms on the multiparameter monitor. Cardiac output (CO) measurements were obtained by bolus thermodilution using a cardiac monitor (Vigilance, Baxter Healthcare Corporation). The cardiac index (CI) was calculated according to the formula k. BW^2/3^, where k = 0.09. The heart rate (HR) and rhythm, mean arterial pressure (MAP), central venous pressure (CVP), mean pulmonary artery pressure (MPAP) and pulmonary artery occlusion pressure (PAOP) were recorded directly from the multiparameter monitor (IntelliVue MP40, Phillips, Böblinger, Germany). Arterial and mixed venous samples were taken simultaneously for gas analysis (ABL 555, Radiometer, Denmark). The systemic vascular resistance index (SVRI), pulmonary vascular resistance index (PVRI), systemic oxygen delivery index (DO_2_I), systemic oxygen consumption index (VO_2_I) and systemic oxygen extraction ratio (O_2_ER) were calculated using standard formulae [14].

After monitoring, the animals were randomly assigned to 1 of 4 groups (6 animals/group) using closed envelope technique:

- Sham: anesthetized and ventilated.
- ANH15%: submitted to ANH to the target hematocrit of 15%.
- ANH10%: submitted to ANH to the target hematocrit of 10%.
- Hypoxia (Hx): a hypoxia-positive control group ventilated with 6% FiO_2_ for 60 minutes with a hypoxic mixture of oxygen and nitrogen. An oxygen analyzer (Ohmeda, Louisville, USA) positioned at the inspiratory line ensured the consistency of the inspired mixture.

The animals in groups Sham, ANH15% and ANH10% were ventilated with 40% FiO2 during the experiment.

The ANH procedure was performed over a 30-minute period as described previously [15]. Briefly, blood was simultaneously withdrawn and replaced with 6% hydroxyethyl starch (130/0.4, Fresenius) at a 1∶1 ratio. Solutions were warmed to 38°C prior to infusion.

The volume of blood removed (V) was calculated according to the following formula: V = EBV×H0−Ht/Hav, where *EBV* corresponded to the estimated blood volume (80 mL. Kg^−1^), *H0* to the initial hematocrit, *Ht* to the target hematocrit and *Hav* to the average hematocrit ((H0+Ht)/2).

After completion of volume exchange, animals were evaluated for 90 minutes. Episodes of mean arterial pressure diminution to >20% of the baseline were treated with additional fluid (lactated Ringer’s solution). Data were collected before ANH (baseline), immediately after (zero) and at 60 and 90 min after hemodilution ended.

At the end of experiment the anesthesia was deepened, potassium chloride was injected into the animals, and fragments of the frontal cortex and hippocampus were collected. The cerebral cortex samples were processed for total and subcellular fraction (nuclear and mitochondrial) protein extraction. The hippocampi were processed to obtain protein from the cytosolic fraction.

### Cerebral cortex total protein extraction

Approximately 0.5 grams of the cerebral cortex were homogenized in a lysis buffer containing 1% protease inhibitor cocktail (Sigma), 1% NP40 (Sigma), 0.5% sodium deoxycholate (Sigma), 1 mM EDTA (Sigma) and 1 mM EGTA (Sigma) diluted in phosphate-buffered and centrifuged (14,000 rpm) for 20 min at 4°C. The supernatants were transferred into new tubes and stored at −70°C until use. The total protein amount was determined using the Bradford method [16].

### Isolation of cortical subcellular nuclear and mitochondrial fractions

Neuronal nuclear and mitochondrial fractions were isolated using the method described by Booth et al. [17] and modified by centrifugation on discontinuous Ficoll gradients.

The first homogenization step was performed using four grams of frontal cortical tissue homogenized in a Dounce-type glass homogenizer (200 µm clearance) in 15 volumes of homogenization medium (23 mM manitol, 7.4 mM sucrose, 0.25 mM EGTA, 0.075 mM EDTA, 0.1% BSA and 0.5 mM Hepes, Sigma) added to 0.001% digitonin and centrifuged at 850 g for 15 min. The resulting pellet was resuspended in 3 volumes of homogenization medium and centrifuged at 1,500 g for 12 min to obtain the nuclear pellet. The centrifugation supernatant from the first step was added to the second centrifugation supernatant and centrifuged at 9,000 g for 15 min. The pellet was resuspended in 1 volume of homogenization medium and centrifuged at 12,000 g for 15 min to produce the crude mitochondrial pellet. To purify the mitochondria, the pellet was suspended in 2.5 mL of homogenization medium, mixed with 12.5 mL of 12% Ficoll solution and placed at the bottom of an ultracentrifuge tube. Ten milliliters of 7% Ficoll solution was layered over it, followed by 10 mL of homogenization medium. The gradient was centrifuged at 100,000 g for 30 min. The mitochondrial pellet was washed twice and suspended in homogenization medium.

The protein extraction from these fractions was performed according to an adapted protocol from Mishra and colleagues. [10], [11] The mitochondrial and nuclear preparations were homogenized with a modified RIPA buffer (50 mM Trisma-HCl, pH 7.4; 1 mM EDTA; 150 mM NaCl; 1% NP-40; 0.25% sodium deoxycholate; 1 mM PMSF; 1 mM Na_3_VO_4_; 1 mM NaF and 1 µg/ml each of aprotinin, leupetin and pepstatin, Sigma) and centrifuged (14,000 rpm) for 20 min at 4°C. The supernatants were transferred into new tubes and stored at −70°C until use.

The mitochondrial and nuclear proteins were quantified using the Bradford method [16].

### Isolation of the Hippocampal Cytosolic Fraction

The hippocampal tissue was homogenized in a Dounce-type glass homogenizer in 15 volumes of medium containing 0.32 M sucrose, 10 mM Trisma-HCl (pH 6.8) and 1 mM MgCl_2_ and centrifuged at 850 g for 10 min. The supernatant was centrifuged at 100,000 g, 0 to 4°C, for 60 min to obtain the cytosolic fraction. The cytosolic protein amount was determined using the Bradford method [16].

### Western blot analysis of the pro-apoptotic (Bax) and anti-apoptotic (Bcl-x) proteins in the cortex total protein fraction, mitochondria and nuclear fraction

The protein samples (120 µg) were separated on 12% sodium dodecyl sulfate (SDS) polyacrylamide (Bio-Rad, USA) using an electrophoresis gel. Proteins were transferred onto PVDF membranes (Bio-Rad, USA) for 1 hour in 100 V. After 1 hour of blocking with 5% milk in Tris-buffered saline-Tween (TBS-T), the membrane was incubated at room temperature for 1 hour with a rabbit antibody to Bax (1∶300 in 3% milk/TBS-T; N-20, Santa Cruz Biotech, USA) or a mouse antibody to Bcl-x (1∶500 in 3% milk/TBS-T, Millipore, USA). Membranes were washed twice for 10 minutes in TBS-T and incubated to room temperature for 1 hour with IgG-ECL anti-rabbit or anti-mouse conjugated secondary antiserum (1∶10,000–1∶6,000, respectively; Rockland, USA). Blots were washed twice with TBS-T and once with TBS. After the final washes, the membranes were incubated with Chemiluminescence HRP substrate (Millipore, USA) for 1 min. The membrane was exposed to an X-ray film for imaging (Hyperfilm ECL, Amersham Biosciences, USA) to visualize the protein band.

Blots were then stripped, blocked and incubated with the rabbit antibody to β-tubulin III (1∶30,000 in 3% milk/TBS-T, Sigma) for 1 hour at room temperature and developed as previously described.

The films were scanned (HP Scanjet G4000 series) and Bax and Bcl-x protein levels were quantified by densitometry using the computer-assisted image analyzer, ImageJ software (version 1.43 µ, National Institute of Health, USA). Density normalization was achieved by dividing the proteins by β-tubulin III density values.

### Western blot analysis of the NeuN protein in the cortex total protein fraction

Cortical total protein NeuN immunoblotting was performed using a mouse antibody to NeuN (1∶1,000 in 3% milk/TBS-T; Millipore, USA) as described above.

### Determination of Caspase-3 and Caspase-9 activity in the cytosolic fraction of the hippocampus and in the mitochondrial fractions of the frontal cortex

The caspase-3 activity of the hippocampal cytosolic and cortical mitochondrial proteins was determined in an assay medium containing 50 mM Tris-HCl (pH 7.0), 0.5 mM Na-EDTA, 20% glycerol, 120 µg protein and 75 µM DEVD-AFC (a specific fluorogenic substrate for caspase-3; Enzo Life Sciences, Plymouth Meeting, USA). The activity was measured according to the fluorescence of 7-amino-4-trifluoromethylcoumarin released for 5 minutes at 30 s intervals using a spectrofluorometer (Spectra MAX Gemini, Molecular Devices, USA) at 460 nm and a 380 excitation wavelength at 37°C [10], [18].

The caspase-9 activity was determined using 75 µM LEHD-AFC as the fluorogenic substrate (Enzo Life Sciences, USA) as described above.

### Determination of Cortical Nuclear and Mitochondrial DNA Fragmentation

To isolate the nuclear and mitochondrial DNA, cortical nuclear and mitochondrial fractions (50 µL) were added to 630 µL of lysis buffer (1 M Tris-HCl, pH 8.0; NaCl; 0.2 M EDTA, pH 8.2), 0.5% dodecyl sulfate and fresh proteinase K and incubated for 30 min at 55°C. Extraction was performed by adding Tris-buffered phenol, after which the mixture was gently homogenized and centrifuged (14,000 rpm for 15 min); this step was repeated once. Next, phenol-chloroform (1∶1) was added, mixed by inversion and centrifuged (14,000 rpm for 15 min). The aqueous phase was added to 3 M sodium acetate (pH 6.0) and 100% ethanol, gently homogenized and precipitated at −20°C for 30 min. The DNA was centrifuged for 20 min at 14,000 rpm. The pellet was washed twice with 70% ethanol, air dried and suspended in 50 µL of 10 mM Tris (pH 8.0) and 1 mM EDTA. The DNA was quantified using a spectrophotometer (ND-1000, NanoDrop Technologies, USA) [19]. The nuclear and mitochondrial DNA (500 ng) were separated in an 1% agarose gel containing ethidium bromide using electrophoresis [19]. The DNA integrity was observed under UV light exposure. The gels were photographed using a Quantum-ST4-1000/26 M system. The pictures were analyzed with ImageJ software (National Institute of Health).

### Statistical Analysis

The hemodynamic data were analyzed within and among groups using analysis of variance (ANOVA) for repeated measures. Neuronal apoptosis precursors were compared between groups using one-way ANOVA. When appropriate, post-hoc Tukey’s multiple comparison analysis was performed (SigmaStat 11, Systat Software Inc., San Jose, USA; GraphPad Prism 5), and p<0.05 was considered statistically significant.

## Results

All animals survived during the experiment and were included in the hemodynamic and neuronal analysis.

**Figure 1:**
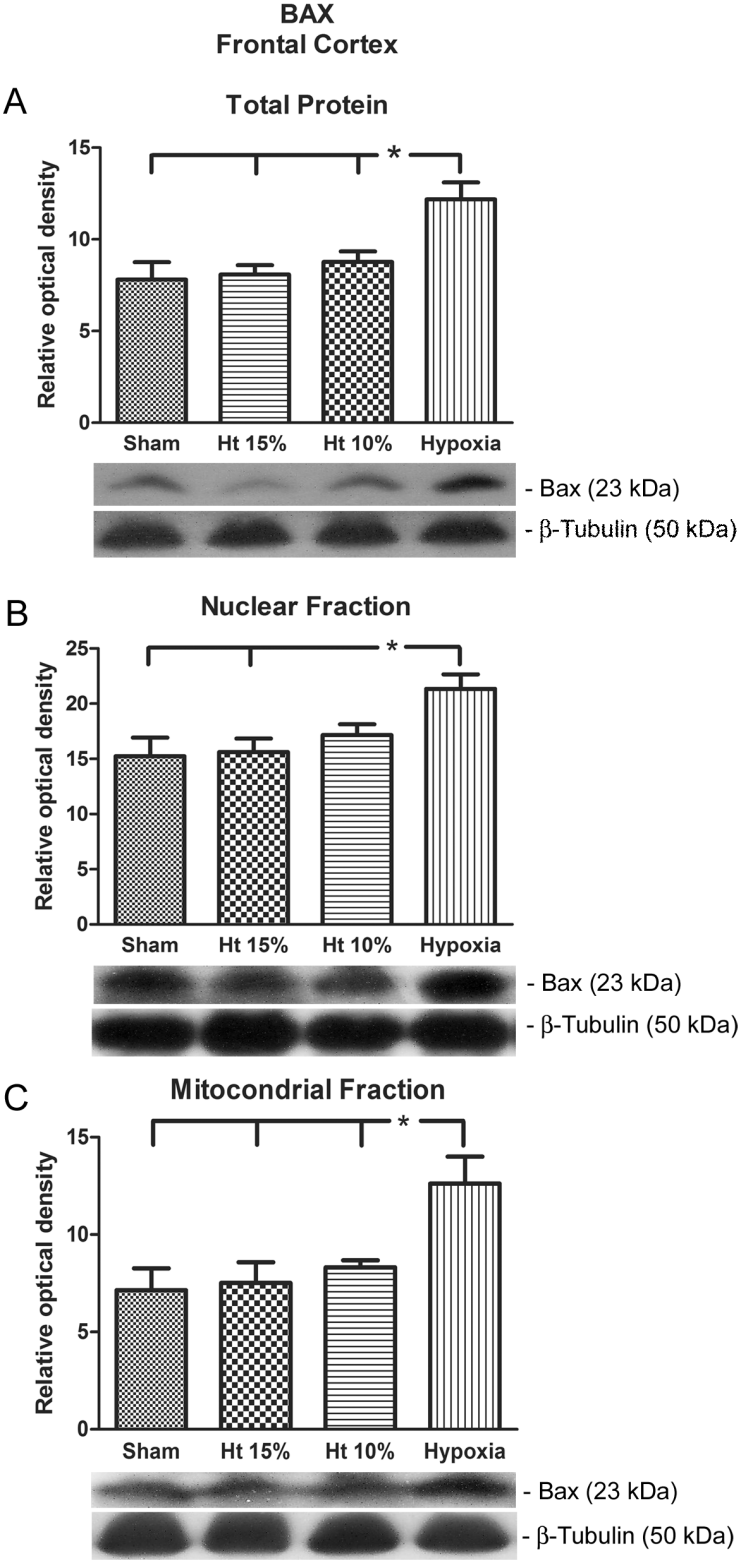
Western blot analysis. The relative optical density of the pro-apoptotic protein Bax in the total protein extraction (A) and the neuronal nuclear (B) and mitochondrial (C) fractions from the cerebral frontal cortex of pigs in the sham, 10% hematocrit (Ht10%), 15% hematocrit (Ht15%) and hypoxia groups (n = 7 per group). β-Tubulin III was used as sample loading control. The representative bands of the Bax (23 kDa) and β-Tubulin III (50 kDa) of animals are illustrated. One-way analysis of variance (ANOVA) was applied to compare the groups. Data are presented as the means ± SEM. *p<0.05.

Hematocrits of 10 or 15% did not increase frontal cortex Bax expression in either the total protein extraction or the mitochondrial fractions compared to the Sham group. The hypoxia-positive group (Hx) showed a significant increase in frontal cortex Bax compared (p<0.05) to all groups. A significant increase in the nuclear fraction was observed in the Hx group compared to the Sham and 15% hematocrit groups (Figure 1).

**Figure 2:**
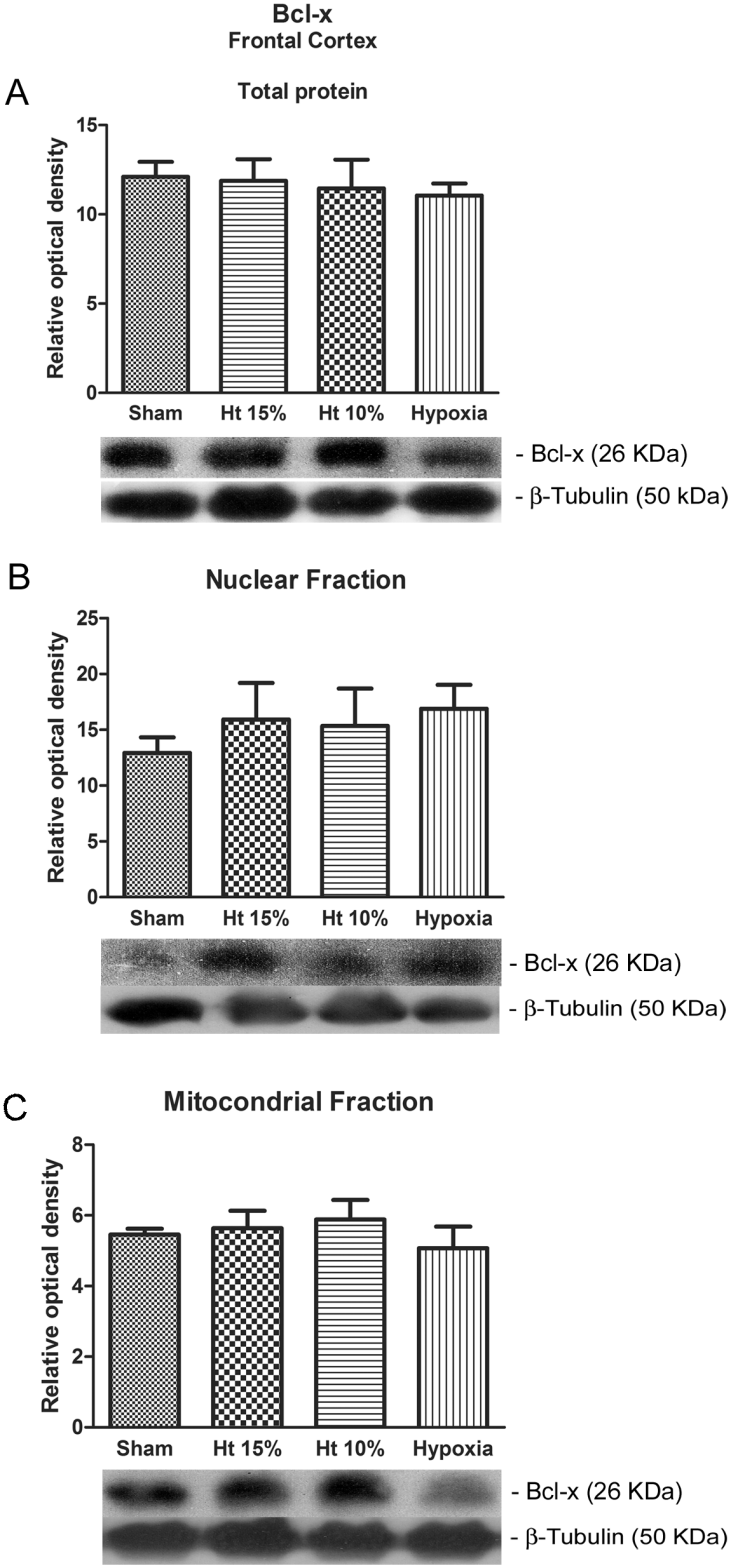
Western blot analysis. The relative optical density of the anti-apoptotic protein Bcl-x in the total protein extraction (A) and the neuronal nuclear (B) and mitochondrial (C) fractions from the cerebral frontal cortex of pigs in the sham, 10% hematocrit (Ht10%), 15% hematocrit (Ht15%) and hypoxia groups (n = 7 per group). β-Tubulin III was used as sample loading control. The representative bands of the Bcl-x (26 kDa) and β-Tubulin III (50 kDa) of animals are illustrated. One-way analysis of variance (ANOVA) was applied to compare the groups. Data are presented as the means ± SEM.

The anti-apoptotic Bcl-x protein expression of the total protein and subcellular fractions did not increase significantly in any of the studied groups (Figure 2).

**Figure 3:**
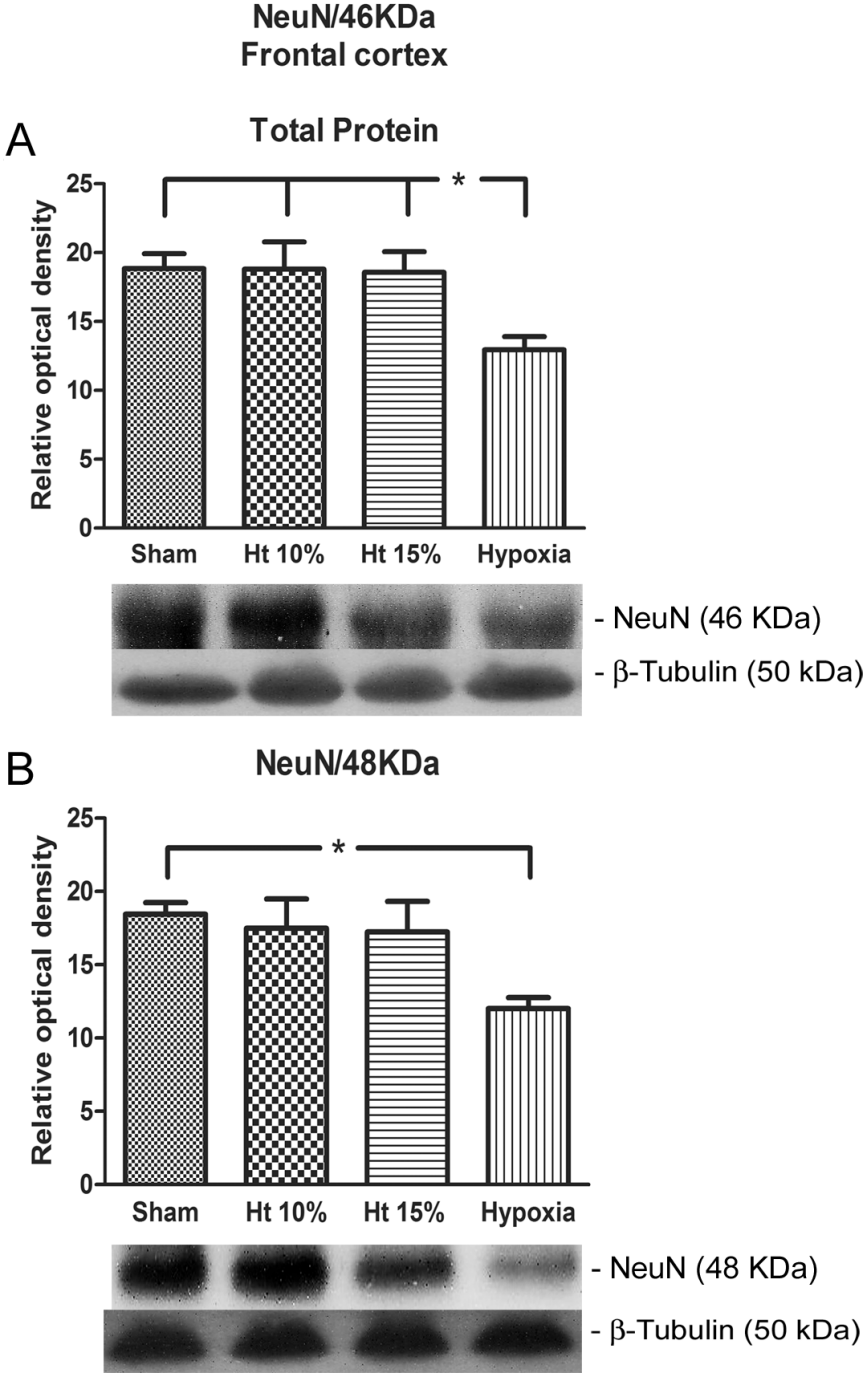
Western blot analysis. The relative optical density of NeuN protein 46 kDa (A) and 48 kDa (B) in the total protein extraction from the cerebral frontal cortex of pigs in the sham, 10% hematocrit (Ht10%), 15% hematocrit (Ht15%) and hypoxia groups (n = 7 per group). β-Tubulin III (50 kDa) was used as sample loading control. The representative bands of the NeuN and β-Tubulin III of animals are illustrated. One-way analysis of variance (ANOVA) was applied to compare the groups. Data are presented as the means ± SEM. *p<0.05.

The anti-NeuN antibody western blot obtained from the frontal cortex total protein extraction showed a significant decrease in the fraction 46 KDa in the Hx group when compared to the other groups, and analysis of fraction 48 KDa showed a significant decrease in the Hx group when compared to Sham group (Figure 3).

**Figure 4:**
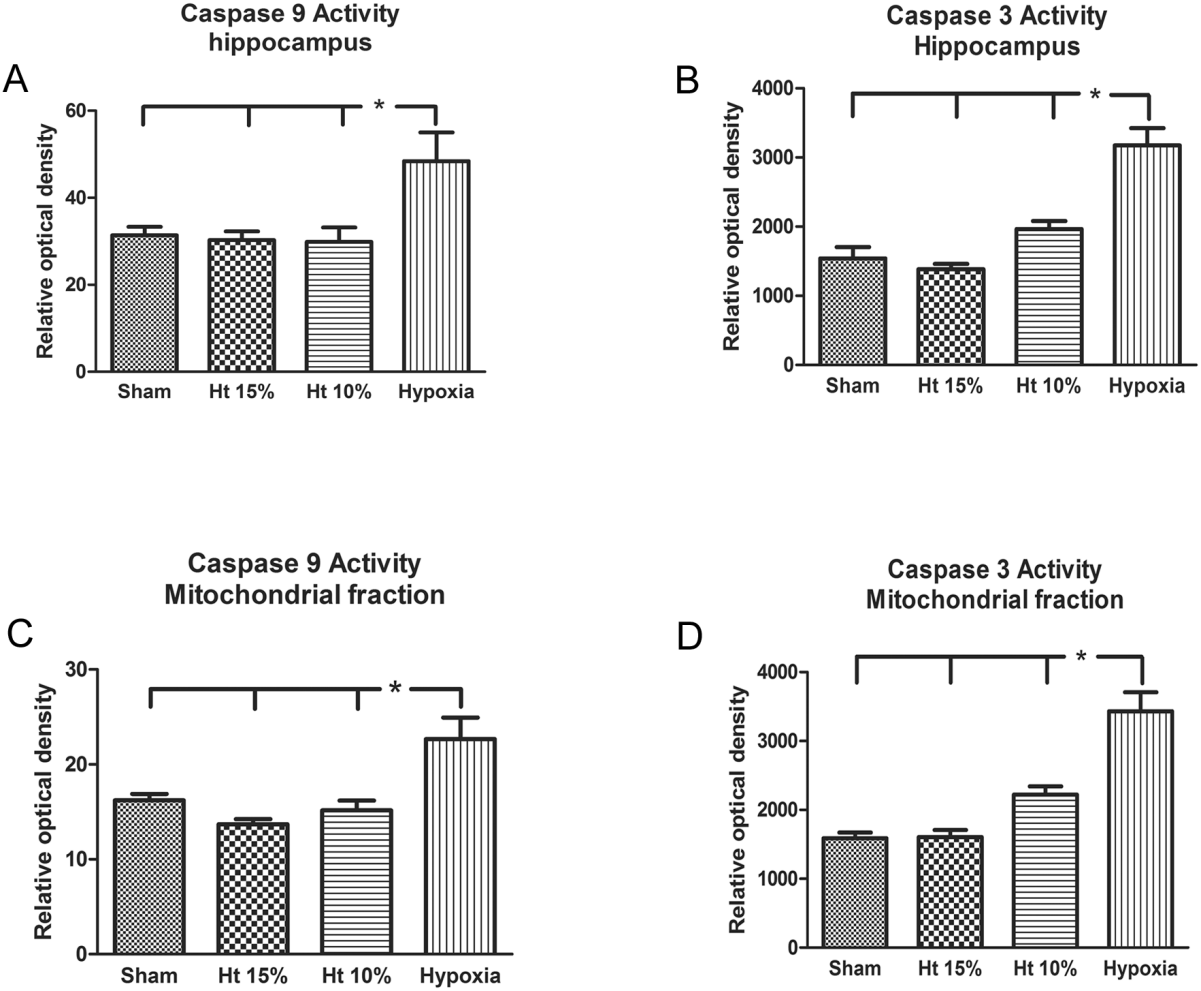
Activity in the cytosolic fraction of caspase 3 and 9 in the cytosolic fraction of the hippocampus (A, B) and the mitochondrial fraction from the cerebral frontal cortex (C, D) of pigs in the sham, 10% hematocrit (Ht10%), 15% hematocrit (Ht15%) and hypoxia groups (n = 7 per group). Data are presented as the means ± SEM. *p<0.05.

Caspase-3 and -9 activity in hippocampus cytosolic and cortical mitochondrial fractions showed a significant increase in the Hx group compared to other groups (p<0.05) (Figure 4).

**Figure 5:**
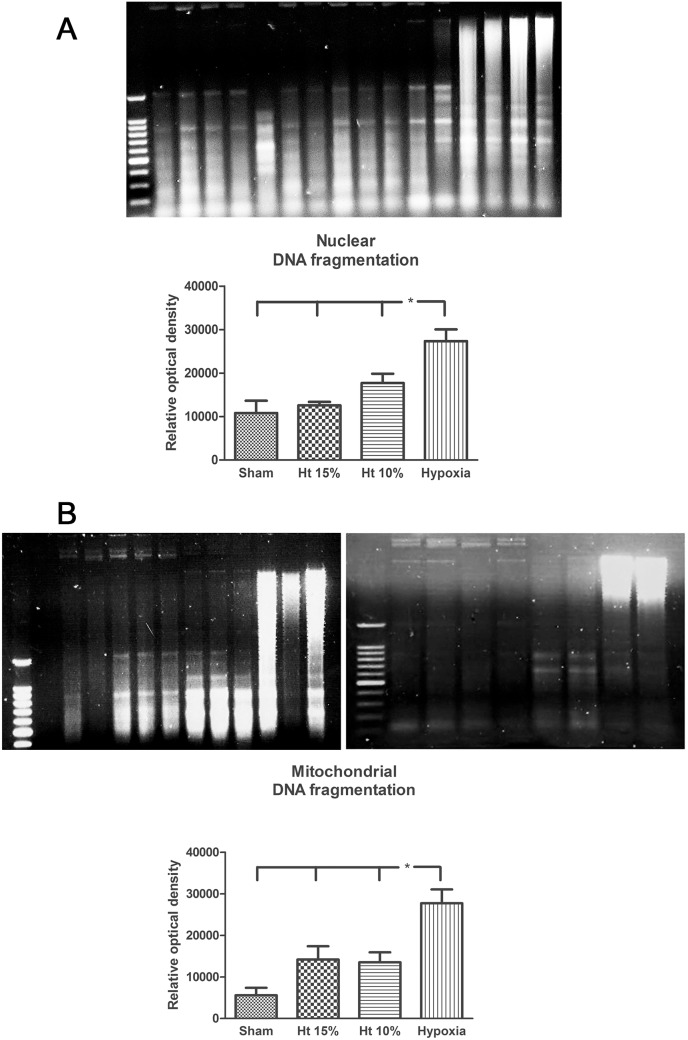
Representative gel electrophoresis and quantitative density images of nuclear (A) and mitochondrial (B) DNA fragmentation in the cerebral frontal cortex of pigs in the sham, 10% hematocrit (Ht10%), 15% hematocrit (Ht15%) and hypoxia groups (n = 4 per group). Data are presented as the means ± SEM. *p<0.05.

Analysis of quantitative density of nuclear and mitochondrial DNA fragmentation showed that ANH10% and ANH15% groups did not differed from Sham group while a significant increase in the Hx group was observed compared to the other groups (p<0.05; Figure 5).

The hemodynamic data showed the compensatory response of hemodilution. The cardiac index and mean arterial pressure increased significantly after hemodilution in the 10 and 15% groups (table 1). Despite a significant decrease in DO_2_, VO_2_ did not change significantly. SvO_2_ remained above 70%, and the lactate level did not increase during the observation period.

**Table 1:** Oxygenation and hemodynamic parameters.

Parameters	Group	T0	T1	T2	T3	Statistics (ANOVA)
DO_2_I ml. min^−1^. m^2^	Sham	583±110	619±6.2	636±70	640±79	Group×time interaction p<0.001
	ANH15%	569±89	509±98^a^	440±76* a	432±61* a	Time p<0.002
	ANH10%	524±141	407±72* a	393±1* a	396±82* a	Group p<0.001
VO_2_I ml. min^−1^. m^2^	Sham	128±20	117±11	125±13	124±10	Group×time interaction p = 0.290
	ANH15%	130±29	134±21	127±16	128±11	Time p = 0.395
	ANH10%	120±40	124±35	103±40	130±16	Group p = 0.481
Arterial lactate mmol/L	Sham	1.5±0.6	1.7±0.6	1.5±0.5	1.6±0.6	Group×time interaction p = 0.153
	ANH15%	1.4±0.3	1.2±0.3	1.2±0.4	1.1±0.2	Time p = 0.320
	ANH10%	1.5±0.4	1.5±0.4	1.5±0.4	1.4±0.4	Group p = 0.150
MAP mmHg	Sham	69±4	72±4	79±7*	76±5	Group×time interaction p<0.001
	ANH15%	75±7	87±7* a	75±7	74±7	Time p<0.001
	ANH10%	74±8	86±6* a	73±6	69±10	Group p = 0.420
CI l/min./m^2^	Sham	3.9±0.6	4.3±0.2	4.6±0.5	4.5±0.6	Group×time interaction p<0.001
	ANH15%	4.1±0.4	7.6±0.9* a	6.0±1.0* a	5.6±0.8* a	Time p<0.001
	ANH10%	3.6±0.8	7.9±1.5* a	6.5±1.4* a	6.1±1.0*	Group p<0.001
SvO_2%_	Sham	78.9±6.3	83.3±2	82.9±1.8	83±1.8	Group×time interaction p<0.019
	ANH15%	79.2±5.5	79.1±1.6	74.8±3.9^a^	74.1±3.3^a^	Time p = 0.064
	ANH10%	81.5±10.4	77.3±5.9	78.7±10.3	71.1±.5* a	Group p<0.004

DO_2_I: Oxygen delivery index; VO_2_I: Oxygen consumption index; MAP: Mean arterial pressure; CI: Cardiac index; SvO_2_: Mixed venous oxygen saturation; T0: baseline; T1: immediately after hemodilution; T2: 60 minutes after hemodilution; T3: 90 minutes after hemodilution.

*P<0.05 different from T0;

a p<0.05 different from Sham group. Data are presented as the means ± SD.

## Discussion

We evaluated the effects of acute anemia on cerebral tissue through the expression of neuronal apoptosis markers in different subcellular fractions in a model of ANH. The cortical and hippocampal regions were evaluated in this study due to their relationship to cognitive function and memory [12]. The results showed that neither 10 nor 15% hematocrit caused hypoxia-induced apoptosis, as demonstrated by the unchanged expression of Bax, Bcl-x and caspase-3 and -9 activity and the absence of DNA fragmentation.

It is interesting that increased levels of pro-apoptotic proteins (Bax) in relation to anti-apoptotic proteins (Bcl-x) and fragmentation changes as a surrogate for apoptosis were only demonstrated by the hypoxia group, while the hematocrit 10 and 15% groups did not demonstrate any change. During hemodilution and anemia, increased blood flow is preferentially directed to cerebral circulation to guarantee brain oxygen transport. The cerebral blood flow increases in proportion to the degree of anemia, as demonstrated in clinical [20] and laboratory studies [6]. This, along with other compensatory mechanisms, guarantees adequate cerebral oxygen tension during acute hemodilution until oxygen consumption becomes supply-dependent [21]. Although cerebral blood flow was not measured in our study, we can infer its preservation as oxygen delivery was maintained throughout the study. An increase in the oxygen extraction rate was observed in the hemodiluted groups, with maintenance of VO_2_I despite a significant decrease in DO_2_I that most likely contributed to oxygen supply adequacy to neuronal cells at this level of anemia. This mechanism can explain why hematocrits of 10 or 15% did not increase the Bax expression in the total frontal cortex, neuronal nuclei or mitochondrial fractions compared to the Sham group.

Low levels of hematocrit during hemodilution has been accepted during the perioperative period because of its short duration and low oxygen consumption during anesthesia. [22] This corroborates our results, as hemodilution lasted 1.5 hours and the 10% hematocrit was safe for cerebral tissue. We do not know if an increased observation time would provoke a different result. The present investigation intended to mimic a situation likely to occur in certain surgical situations where a 10–15% hematocrit level could be acceptable for a short period of time due to the transfusion trigger guidelines strongly recommending transfusions with a hematocrit level below 21% [23].

The augmented pro-apoptotic/anti-apoptotic protein ratio (Bcl-x) in the mitochondrial fraction can change mitochondrial membrane permeability and the apoptotic protease activating factor-1-mediated activation (Apaf-1) of procaspase 9 in caspase 9 that subsequently activates procaspase 3 in caspase 3, which is responsible for cellular death [24]. This is in accordance with our results, as expression of caspase 3 and 9 was augmented in both the cytosolic hippocampus and mitochondrial fractions in the Hx group. Other experimental studies have shown similar results [10], [18], [25]. The absence of changes in the Bax/Bcl-x ratio and in the activity of caspase 3 and 9 in the hemodiluted groups are interesting because, to our knowledge, no other study has evaluated the presence of these apoptotic markers at 10 and 15% hematocrit levels in cerebral tissue. However, it cannot be ruled out that more intense anemic conditions for a longer period of time could result in apoptosis and lead to alterations in Bcl family protein expression.

Our results contradict other experimental studies focused on hemodilution that found increased cerebral expression of hypoxia to induce markers such as HIF-1α and neuronal nitric oxide synthase [6]. However, these studies have not shown the association between these markers and neuronal injury. Other studies have also demonstrated that hemodilution can exacerbate neurological injury after CPB with or without circulatory arrest [12], [26], [27], focal cerebral ischemia [28] and trauma [29]. Nevertheless, these results demonstrate the sum effect of hemodilution and CBP, not the effect of isolated acute anemia. Furthermore, our animals were maintained within a normal temperature range, thus avoiding cerebral protection by hypothermia.

Our results also showed that while hypoxia significantly increased mitochondrial DNA fragmentation in the Hx group, there was no increase with hemodilution to target hematocrits of 10 or 15%. The apoptotic nuclear DNA fragmentation process associated with cerebral hypoxia is initiated by activation of nuclear Ca^2+^ influx mechanisms and increases transcription of a number of genes, including the apoptotic genes of the Bcl-2 family responsible for programmed cell death. Proapoptotic proteins initiate apoptosis by activating a caspase system cascade that leads to cleavage of numerous intracellular proteins responsible for the maintenance of cell structure and results in chromosomal DNA cleavage and disruption of cell nuclei [30].

In addition to the previously cited neuronal markers, we evaluated anti-NeuN antibodies using western blots of the total protein in the frontal cortex, which was significantly decreased in the Hx group and unchanged in the Control, ANH10% and ANH15% groups. NeuN is expressed almost exclusively in the nervous system. It appears at the beginning of development and persists until adulthood, and its solely by neurons makes NeuN a specific marker for neuronal nuclei [31]. NeuN expression is reduced in several pathological conditions affecting neuronal viability such as cerebral ischemia, hypoxia and trauma [32], [33]. Our results suggest that hematocrits as low as 10% do not induce a reduction in the number of neuronal nuclei at this early period of evaluation, corroborating the absence of mitochondrial fragmentation and apoptosis in these groups.

One limitation of this study was the lack of measurements of cerebral oxygenation status and regional blood flow. The duration of anemia (90 minutes) may have been insufficient to induce an increase in apoptosis precursors. Anesthesia may also have contributed by decreasing the cerebral metabolism. Inhaled anesthetics also offered protection against ischemic injuries in various organs [34], [35]. Ventilation with 40% oxygen during the procedure may also have preserved cerebral oxygenation. Meier et al. observed that increasing the FiO2 from 21% to 100% in pigs submitted to 10% ANH, reduced the 6-h mortality rate from 100% to 14% [36]. Another limitation was the inability to isolate nuclear and mitochondrial fractions due to the limited material from hippocampus, and thus, only cytosol caspase-3 and -9 activity was measured.

## Conclusion

Acute normovolemic hemodilution to the target hematocrits of 10 and 15% did not increase expression of apoptosis precursors in cortical or hippocampal tissue during the acute phase. Alternatively, acute hypoxia provoked neuronal alterations.
